# A successful hybrid deep learning model aiming at promoter identification

**DOI:** 10.1186/s12859-022-04735-6

**Published:** 2022-05-31

**Authors:** Ying Wang, Qinke Peng, Xu Mou, Xinyuan Wang, Haozhou Li, Tian Han, Zhao Sun, Xiao Wang

**Affiliations:** Systems Engineering Institute, Xi’an Jiaotong University, Xi’an, China

**Keywords:** Promoter identification, Convolutional neural networks (CNNs), Fully connected networks, Structural profiles

## Abstract

**Background:**

The zone adjacent to a transcription start site (TSS), namely, the promoter, is primarily involved in the process of DNA transcription initiation and regulation. As a result, proper promoter identification is critical for further understanding the mechanism of the networks controlling genomic regulation. A number of methodologies for the identification of promoters have been proposed. Nonetheless, due to the great heterogeneity existing in promoters, the results of these procedures are still unsatisfactory. In order to establish additional discriminative characteristics and properly recognize promoters, we developed the hybrid model for promoter identification (HMPI), a hybrid deep learning model that can characterize both the native sequences of promoters and the morphological outline of promoters at the same time. We developed the HMPI to combine a method called the PSFN (promoter sequence features network), which characterizes native promoter sequences and deduces sequence features, with a technique referred to as the DSPN (deep structural profiles network), which is specially structured to model the promoters in terms of their structural profile and to deduce their structural attributes.

**Results:**

The HMPI was applied to human, plant and Escherichia coli K-12 strain datasets, and the findings showed that the HMPI was successful at extracting the features of the promoter while greatly enhancing the promoter identification performance. In addition, after the improvements of synthetic sampling, transfer learning and label smoothing regularization, the improved HMPI models achieved good results in identifying subtypes of promoters on prokaryotic promoter datasets.

**Conclusions:**

The results showed that the HMPI was successful at extracting the features of promoters while greatly enhancing the performance of identifying promoters on both eukaryotic and prokaryotic datasets, and the improved HMPI models are good at identifying subtypes of promoters on prokaryotic promoter datasets. The HMPI is additionally adaptable to different biological functional sequences, allowing for the addition of new features or models.

**Supplementary Information:**

The online version contains supplementary material available at 10.1186/s12859-022-04735-6.

## Introduction

According to their definition in genetics, functional areas around transcription start sites (TSSs), which are crucial to initiating and regulating DNA transcription are referred to as promoters [1, 2]. Therefore, reliable promoter identification is a crucial course of action in genomics for furthering our present understanding of genetic regulation networks.

Researchers face a significant barrier in identifying promoters and nonpromoters, such as human and plant promoters, that have a greater variety and are more difficult to describe [3, 4]. In recent years, a variety of biological experimental base approaches have been used to find promoters [5]. Such traditional procedures were both difficult and costly [6]. Newer promoter identification methods based on computational techniques with lower complexity and running costs have recently been proposed [7]. Therefore, determining the difference between nonpromoters and promoters and extracting the most distinctive characteristics for promoter recognition among various species is critical. Context features, signal features and CpG features are the three types of traits employed to characterize promoters in general. Transcription factor recognition elements [8], CAAT boxes [9], TATA boxes [10] and other functional promoter element regions are always used to extract signal features. Consequently, a number of other regions of the promoter are discarded. Context features are obtained by executing k-length windows and estimating the k-mer (plausible subsequences of length k) frequency [11]. Nonetheless, certain information, such as the spatial connections among the base pairs placed within the sequences, has still been overlooked. The existence of CpG islands was used for the identification of promoter regions in CpG feature-based approaches [12, 13]. However, because CpG islands are contained within only 70% of promoters, the methods are unlikely to significantly enhance the identification outcome. To conclude, the techniques comprising three single features are frequently insufficient.

Furthermore, novel sequence feature-based techniques for promoter identification have recently been proposed and have yielded promising results. Successful identification of promoter areas was made by Umarov et al. (2017) using primary sequences of promoters without any prior understanding of certain promoter properties [14]. To predict the strength of Escherichia coli promoters, Bharanikumar et al. used position weight matrices to represent the promoter sequences [15]. These findings suggest that the primary sequences of promoters may imply more information on discriminative factors than these aforementioned traits. However, because promoters are typically complicated and heterogeneous, promoter sequence-based signals cannot reliably identify promoters very well. According to recent research, structural characteristics have a key role in a variety of bioprocesses [16]. Although DNA is frequently represented as a rather inflexible double-helical structure, the innate structural attributes provide a wealth of useful details [17]. Whereas the nucleotide sequence mostly determines these structural features, research has demonstrated that promoters do possess different patterns in terms of their structure compared to other sequences [18]. This outcome suggests that the structural attributes that indicate promoter structural profiles have the potential to be employed as a supplement to the primary sequences in promoter identification.

The focus of such research has always been the identification methods and models, in addition to the methods describing promoter properties. Deep neural networks have recently been used in tasks such as promoter identification and recognition, owing to the remarkable performance and excellent application of deep learning models in different sectors. Convolutional neural networks (CNNs) were utilized for the analysis of the sequence characteristics of eukaryotic and prokaryotic promoters and for building prediction models by Umarov et al. [14]. In addition, Oubounyt et al. postulated the DeePromoter model for examining and analysing the essential features of the sequences of short eukaryotic promoters and for the accurate recognition of the promoter sequences of mice and humans [19]. The DCDE deep learning method for the extraction of distinctive characteristics from human promoters was proposed by Xu et al. [20].

For better modelling of the promoters and improvement in the identification outcomes, we developed a hybrid model for promoter identification (HMPI) aimed at identifying the promoter. The HMPI is in fact inspired by these aforementioned studies and the outstanding modelling potential of deep learning-based algorithm. We propose the PSFN (promoter sequence features network) method to model the original promoter sequences and derive the sequence features based on CNNs. Additionally, in the PSFN, we incorporate the centre loss as an aspect of the classification loss function to further boost the specificity of promoters and nonpromoters. The validity of the HMPI is demonstrated by the identification results using the primary promoter sequences as input. Furthermore, to model the promoter structural profiles and extract structural features, we propose the DSPN (deep structural profiles network), which contains smaller connections among layers, based on a fully connected network and DenseNet [21]. Owing to the DSPN layers being directly connected, the network can be much deeper, more efficient, and more precise for modelling promoter structural characteristics. Ultimately, we build the HMPI, which combines the DSPN and PSFN. The efficiency of the HMPI was demonstrated by experiments on datasets corresponding to both plants, humans and the Escherichia coli K-12 strain.

The main contribution of the present research is the advancement of an effective hybrid deep learning model called the HMPI for promoter identification. In the HMPI, the original sequences and structural profiles of promoters are modelled simultaneously through the PSFN and DSPN, which are methods we proposed based on CNNs, fully connected networks and DenseNet. Additionally, instead of deriving a single type of feature, we extracted and combined the sequence features and structural features for promoter identification. The experimental results demonstrate that the HMPI can significantly improve the promoter identification performance on both eukaryotic and prokaryotic promoter datasets. The results also suggest that the structural information recovered by the DSPN and the distinguishing element information extracted by the PSFN may complement one another in promoter identification. In addition, after the improvements of synthetic sampling, transfer learning and label smoothing regularization, the improved HMPI models achieved significant results in identifying subtypes of promoters on the subdatasets of prokaryotic promoters. Furthermore, as a hybrid model, the HMPI can be extended to include more characteristics and has the prospects of application to various functional biological sequences.

## Experiments and results

A variety of experiments are presented in this section to provide evidence of the efficacy of our approaches and models. We used Keras, which is a Python-based approach, to conduct the experiments (https://keras.io/).

### Data preparation and performance assessments

The datasets used in the investigation were gathered from eukaryotes and prokaryotes. To identify eukaryotic promoters, we collected promoter datasets from both plants and humans. To obtain sufficient promoter data to conduct the experiments, the Eukaryotic Promoter Database (EPD) was searched, and all the cases of 29,597 human promoters were collected [22]. Furthermore, PlantPromDB was searched and all the cases of 8272 plant promoters were obtained [23]. These datasets provide verified high quality promoter data. For negative datasets, nonpromoters for humans were collected from the UCSC database (http://www.genome.ucsc.edu), and nonpromoters for plants were processed from TAIR [24]. The negative sequences were gathered from regions such as exons, coding regions, introns, and 3' untranslated regions, and the negative sequence start site was selected randomly based upon the premise that the sequence length of 251 bp was sufficient. Table 1 lists the specifics of these datasets.

**Table 1:** Datasets and the details of eukaryotic promoters

Organism	Data sources	Dataset type	Numbers of sequences	Location/length
Human	EPD	Promoters	29,597	[− 200, + 50] bp
	UCSC	Non-promoters	50,000	251 bp
Plants	PlantProm DB	Promoters	8272	[− 200, + 50] bp
	TAIR	Non-promoters	12,834	251 bp

To identify prokaryotic promoters, the Regulon DB [25] was searched and the data were processed as the same processing flow proposed by Bin et al. [26]. All the cases of 2860 promoters of Escherichia coli K-12 strain were collected. These promoters came from six different subtypes of promoter data, and the negative samples were selected randomly from coding regions of the Escherichia coli K-12 strain. Table 2 lists the specifics of these datasets.

**Table 2:** Datasets and the details of prokaryotic promoters

Organism	Data sources	Subtype/type	Numbers of sequences	Location/length
*Escherichia coli* K-12	Regulon DB	σ24	484	[− 60, + 20] bp
		σ28	134	[− 60, + 20] bp
		σ32	291	[− 60, + 20] bp
		σ38	163	[− 60, + 20] bp
		σ54	94	[− 60, + 20] bp
		σ70	1694	[− 60, + 20] bp
		Non-promoters	2860	81 bp

In this study, all samples are divided according to the same proportion, 1/5 as a test set, 4/5 as a training set. The widely used measures of the sensitivity $Sn$, the specificity $Sp$, the Matthew correlation coefficient $Mcc$ [27], and the accuracy $Acc$ are utilized to assess the performance of models.

### Effectiveness of the PSFN at identifying eukaryotic promoters

The PSFN for modelling the original promoter sequences and deriving sequence features in the HMPI is developed in “Modelling the original promoter sequences” section of “Methods”. The results of the studies in this section are shown to confirm the efficiency of the features derived from promoter sequences through the PSFN. We also compared a method called PSFNcce, which has a model structure that is nearly identical to the PSFN. Only the loss function distinguishes these two approaches. The PSFN employs the joint loss function (Eq. 9) whereas PSFNcce employs the categorical cross-entropy (CCE) loss function (Eq. 8). In these two approaches, the Adam optimizer is employed to optimize both objective functions [28]. In addition, as comparison tests, two more cutting-edge deep learning classification models, GoogLeNet [29] and ResNet [30], are utilized to predict promoters. Table 3 shows the outcomes of using these four approaches to identify the promoters and the negative samples in the test set, on the datasets of humans and plants which are indicated in Table 1.

**Table 3:** Detailed outcomes of the four methods mentioned above

Organism	Method	*Sn* (%)	*Sp* (%)	*Acc* (%)	*Mcc*
Human	PSFNcce	**86.79**	92.32	90.26	0.7930
	PSFN	85.16	**94.42**	**90.97**	**0.8055**
	ResNet	82.13	90.60	87.45	0.7303
	GoogLeNet	83.75	86.84	85.69	0.6982
Plants	PSFNcce	**87.92**	94.62	91.99	0.8314
	PSFN	86.96	**96.96**	**93.04**	**0.8539**
	ResNet	80.92	89.56	86.17	0.7086
	GoogLeNet	87.92	92.83	90.90	0.8089

The best results of each measure are shown in bold

**Fig. 1:**
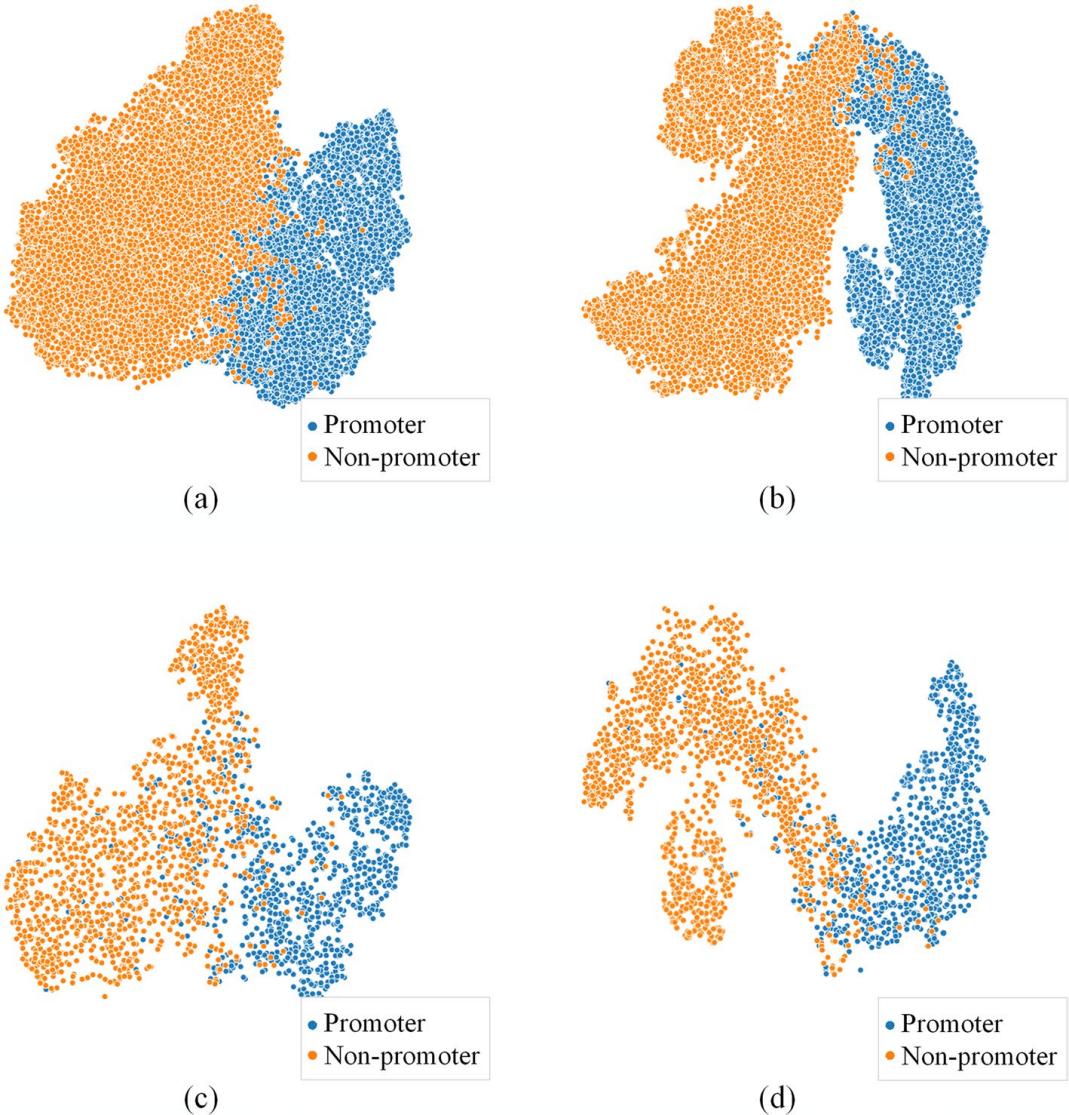
**a** Features derived from the training sets of plants through the PSFNcce. **b** Features derived from the training sets of plants through the PSFN. **c** Features derived from the test sets of plants through the PSFNcce. **d** Features derived from the test sets of plants through the PSFN

Table 3 shows that PSFNcce has higher sensitivity $Sn$ than the other three techniques, implying that it performs better on positive samples (promoter sequences). On both human and plant datasets, the PSFN outperforms the other three approaches in terms of the $Mcc$, $Acc$, and $Sp$. The results demonstrate the efficiency of the PSFN, and the sequence features recovered by the PSFN can be used to characterize human and plant promoters from a variety of angles. As previously stated, the PSFN approach uses the centre loss as a component of the joint loss function to improve the discriminative ability of the learned sequence features. We lower the dimensions by PCA (principal component analysis) and TSNE (t-distributed stochastic neighbor embedding) methods [31] and show the features of the sequences extracted via PSFNcce and the PSFN to check the validity of the centre loss intuitively. The reduction and display of the plant datasets is as an example due to space limits. Figure 1a, b depicts the sequence features retrieved from the training set, and Fig. 1c, d represents the features derived from the test set.

The comparison in Fig. 1 clearly shows that, in the right figures, the data in clusters lie close to each other, and there are clearly fewer interleaved and overlapped parts among the various classes of clusters in the left figures. With the centre loss as a loss function element, the models reveal increased intraclass compactness and interclass dispersion.

### Effectiveness of the DSPN at identifying eukaryotic promoters

We develop the DSPN in “Extraction of the structural characteristics of promoters” section of “Methods” and used the DSPN in the HMPI to model the structure-related profiles and extract the structural features from the primary promoter sequence. The outcomes of the trials for confirming the efficiency of the structural features retrieved with the DSPN will be presented in this section.

The matrices of the structural profile properties derived from the sequences of the promoter through Eq. (11) are used as the inputs of the DSPN to evaluate the capabilities of the DSPN to characterize structural profiles and authenticate the efficiency of the deduced structural features. With a $12 \times( L - 1)$ input of the matrix (see Eq. 11) from a primary sequence of promoters, as discussed in the “Extraction of the structural characteristics of promoters” section of “Methods”, the softmax activation function is employed to allow the possibility of the input sequence belonging to each category, and the CCE loss function (see Eq. 8) is employed as the supervision signal for training the network. Moreover, the comparative experiments utilize a set of three frequently applied deep learning models with comparatively better efficiency. With the same inputs as the DSPN, the CNNs [14], GoogLeNet [29], and ResNet [30] are applied to identify promoters. Table 4 shows the results of the DSPN and three other comparative models with the input of matrix $S$ of promoters and negative samples in the test set, on the datasets of humans and plants, which are indicated in Table 1.

**Table 4:** Detailed outcomes of the four methods mentioned above with the input of matrices of structural profile properties

Organism	Method	*Sn* (%)	*Sp* (%)	*Acc* (%)	*Mcc*
Human	DSPN	80.60	**90.36**	**86.73**	**0.7144**
	CNNs	**82.63**	86.76	85.22	0.6874
	ResNet	80.81	86.84	84.59	0.6725
	GoogLeNet	78.31	89.56	85.37	0.6847
Plants	DSPN	75.24	90.33	**84.41**	**0.6695**
	CNNs	**81.76**	85.50	83.23	0.6607
	ResNet	71.37	90.80	83.18	0.6428
	GoogLeNet	67.51	**93.69**	83.42	0.6500

The best results of each measure are shown in bold

Table 4 shows that the CNNs outperforms the other three models in terms of the $Sn$, and the DSPN outperforms the other three models in terms of the $Mcc$, $Acc$, and $Sp$ on the human dataset, which implies that the DSPN is better at describing the structural profiles of the human promoters of sequences, and the CNNs is better at describing human promoters than nonpromoters. On the plants dataset, Table 4 shows that the DSPN has the best performance on the $Mcc$ and $Acc$, and GoogLeNet and the CNNs have the best $Sp$ and $Sn$. This implies that promoter sequences can be best characterized by CNNs, and nonpromoter sequences can be best characterized by GoogLeNet. Similarly, the plant datasets can be comprehensively characterized by the DSPN.

Comparing Table 3 with Table 4 shows that simply using promoter structural profiles to identify promoters is less successful than using promoter sequences, indicating that original sequences include more discriminative information than generated structural characteristics. Furthermore, the identification results utilizing the sequences of promoters on plant datasets are superior to those on human datasets, as presented in Table 3; however, the opposite is true in Table 4, utilizing the structural profiles of promoters. It can be inferred that the extracted sequence features can be distinctly differentiated from the structural features. The information implied in structural features and the information implied in sequence features may complement each other.

### Effectiveness of the HMPI at identifying eukaryotic promoters

In this part, we will first show the conservative property of the structural profiles of promoter sequences. Statistical analysis is performed on the twelve properties described in Additional file 1: Table S1. Owing to space constraints, we show the statistical results on the plant datasets as a demonstration in Additional file 1: Figure S1. Figure S1 shows that there were obvious differences in the average expression of each structural profile ($SP$) property between plant promoters and nonpromoters. Especially in the core promoter regions near the TSSs (locations at coordinate 0 in Additional file 1: Figure S1), the statistical results of promoters show significant variation in the expression of each $SP$ property while the curves of nonpromoters are relatively flat. This indicates that these twelve $SP$ properties of promoter sequences are strongly conserved and can be used for further feature extraction and promoter identification.

Next, we will compare the performance of the HMPI on plant and human datasets and compare the HMPI to the most sophisticated and elaborate classification models GoogLeNet and ResNet, the promoter identification techniques for humans [20, 32, 33], and the promoter identification techniques for plants [27, 34] that have been recently put forward. SD-MSAEs [32] created a human promoter recognition technique by combining the advantages of several sparse autoencoders and statistical divergence within deep learning. SCS [33] used decision trees to build a hierarchical promoter recognition system that included CpG, k-mer, and structural data. DCDE-MSVM [20] was found to be a highly effective deep convolutional divergence encoding technique based on CNNs and statistical divergence. using a genetic algorithm. PromoBot [34] chose triplet pairs utilizing a genetic algorithm to differentiate between promoters and nonpromoters on the basis of the frequency of nonadjacent triplet pairs and later classified them using an SVM. TSSPlant [27] employed a model based on a backpropagation artificial neural network to predict promoters based on eighteen major signal and compositional properties of plant promoter sequences. Table 5 shows how different strategies compare in terms of performance.

**Table 5:** The comparison of the performance of the HMPI and other methods mentioned above at identifying eukaryotic promoters

Organism	Results source	*Sn* (%)	*Sp* (%)	*Mcc*	Method
Human	This article	**85.84**	**94.72**	**0.8151**	HMPI
		82.13	90.60	0.7303	ResNet
		83.75	86.84	0.6982	GoogLeNet
	[32]	85.19	81.91	$*$	SD-MSAEs
	[33]	78.45	$*$	0.6413	SCS
	[20]	79.67	78.90	*	DCDE-MSVM
Plants	This article	90.34	**95.95**	**0.8684**	HMPI
		80.92	89.56	0.7086	ResNet
		87.92	92.83	0.8089	GoogLeNet
	[34]	89	86	*	PromoBot
	[27]	**94**	86	0.82	TSSPlant

The best results of each measure are shown in bold

^*^The represented measurements are not calculated

Table 5 clearly shows that the HMPI has the best $Mcc$, $Sp$, and $Sn$ results on human datasets. Similarly, the HMPI has also been able to achieve the highest $Mcc$ and $Sp$ on the plant datasets. This suggests that the HMPI is quite good at identifying human promoters. TSSPlant has the best $Sn$ results on the plant datasets, but it also has the lowest $Mcc$ and $Sp$, implying that it has a larger false-positive rate, which suggests that the HMPI continues to have the best overall identification results on the plant datasets. These results authenticate the validity of the HMPI.

### Application of the HMPI to identify prokaryotic promoters and their types

In this section, we will first compare the performance of the HMPI at identifying promoters on the Escherichia coli K-12 strain datasets to those of several identification models [26, 35–37]. Stability [35] used DNA double stranded stability features to identify promoters. iPro54 [36] considered local and global pseudonucleotide composition (PSEKNC) characteristics for promoter identification. iPromoter-2L [26] considered the influence of different sliding windows based on PSEKNC. MULTiPly [37] applied global statistical features to classify promoters. To accommodate the changes in the data length of Escherichia coli K-12 strain promoter sequences, the number of nodes in the DSPN is set to 80, 500, 80, 150, 500, 500, and 128, respectively, in this section. Table 6 shows the outcomes of identifying promoters through the HMPI and four other comparative models on the Escherichia coli K-12 datasets, which are indicated in Table 2.

**Table 6:** The comparison of performance of the HMPI and other methods mentioned above at identifying prokaryotic promoters

Organism	Method	*Sn* (%)	*Sp* (%)	*Acc* (%)	*Mcc*
*Escherichia coli* K-12	HMPI	**90.21**	**88.11**	**89.16**	**0.7834**
	Stability	76.61	79.48	78.04	0.5615
	iPro54	77.76	83.15	80.45	0.6100
	iPromoter-2L	79.20	83.15	80.45	0.6343
	MULTiPly	87.27	86.57	86.92	0.7385

The best results of  each measure are shown in bold

Table 6 shows that compared to the other four methods, the HMPI achieves the best results in all four indices of the $Sn$, $Sp$, $Acc$ and $Mcc$. This indicates that the features derived from the HMPI can better represent the promoter of the Escherichia coli K-12 strain and that the HMPI is well suited for identifying prokaryotic promoters. However, there are always multiple subtypes of prokaryotic promoters. For instance, six subtypes for promoters of the Escherichia coli K-12 strain are indicated in Table 2. We will continue with several experiments to confirm the validity of the HMPI at identifying prokaryotic promoter subtypes in this section.

As illustrated in Table 2, the data volumes of promoters in each subtype vary greatly, and several of the volumes are quite small for modelling. To better adapt to these problems, we made different changes to the HMPI, and the improved HMPIs are denoted as HMPIat and HMPIlsr.

**Fig. 2:**
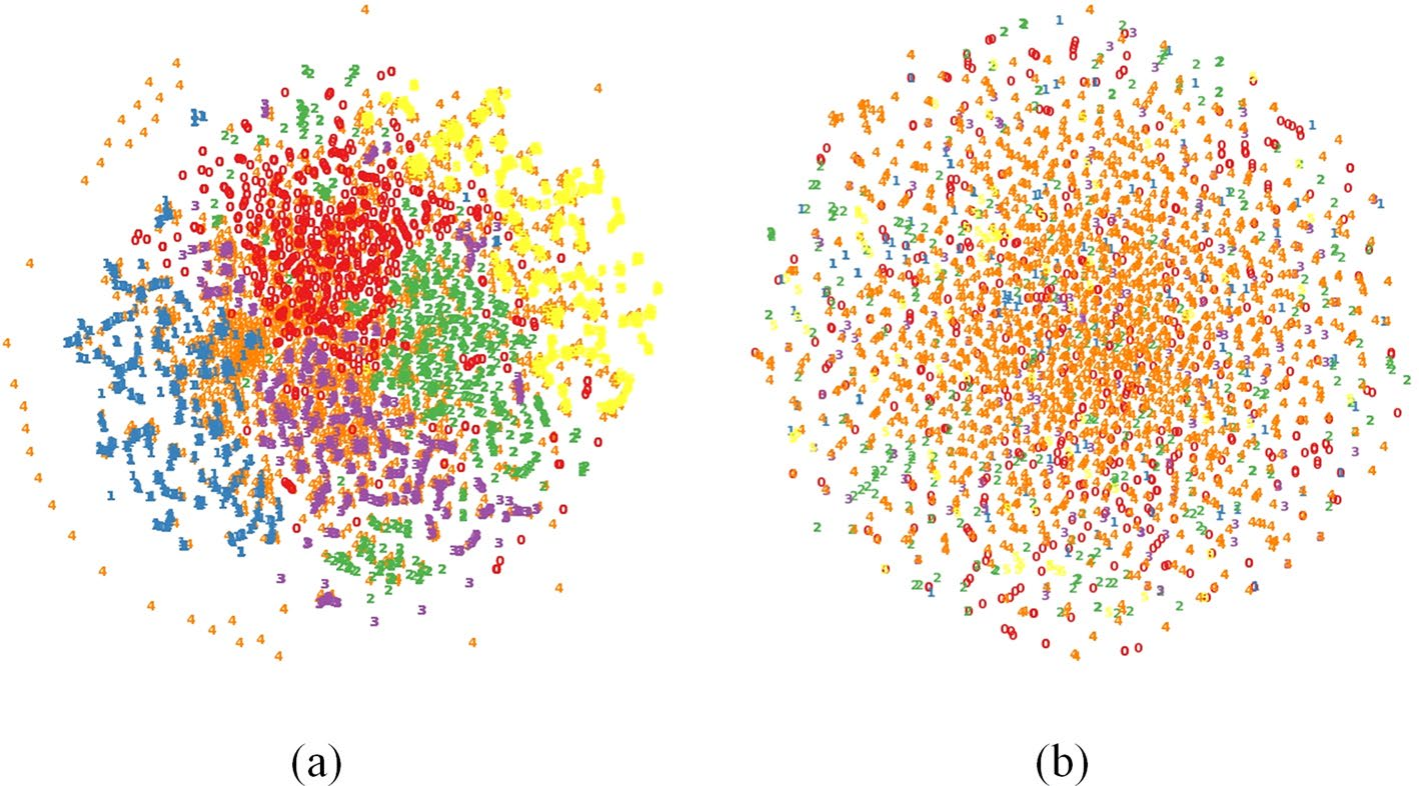
**a** The training set features derived by the second layer of the PSFN within the HMPI. **b** The training set features derived by the second layer of the PSFN within the HMPIat

First, in HMPIat, we introduce an adaptive synthetic sampling approach ADASYN [38] for learning imbalanced datasets, which uses a weighted distribution for different minority class examples according to their level of difficulty in learning and generates more synthetic data for minority class examples. In addition, because the one-hot encoding of the promoter sequence is difficult for adaptive synthetic sampling, we apply transfer learning on the second layer of the PSFN [39] to generate synthetic data. Figure 2a depicts the training set features derived by the second layer of the PSFN within the HMPI, and Fig. 2b depicts the training set features derived by the second layer of the PSFN model within HMPIat, both on the Escherichia coli K-12 dataset. As shown in Fig. 2, the application of the ADASYN and transfer learning makes the distribution of minority classes clearer.

Second, in HMPIlsr, we propose the utilization of a label smoothing regularization (LSR) method to assign a uniform label distribution to the nonground truth classes, which can regularize the supervised model, as inspired by Zhedong et al. [40]. The distribution of the ground truth is denoted by $q(k)$ in Eq. (7), and the distribution of LSR can be denoted as follows.

1 $$q_{LSR} (k) = \left\{ {\begin{array}{*{20}l} {\frac{\varepsilon }{K},k \ne y} \\ {1 - \varepsilon + \frac{\varepsilon }{K},k = y} \\ \end{array} } \right.$$

where $K$ is the number of classes and $\varepsilon$ is a small number. In this paper, we set $\varepsilon$ as $0.2$. Substituting Eq. (1) into Eq. (6), we obtain the categorical cross-entropy loss function with LSR, as shown in Eq. (2). In addition, compared to the HMPI method, Eq. (2) replaces Eq. (6) in the HMPIlsr method.

2 $$L_{LSR} = - (1 - \varepsilon )log(p(y)) - \frac{\varepsilon }{K}\sum\limits_{k = 1}^{K} {\log (p(k))}$$

Finally, we compared the promoter subtype identification performance of the HMPI, HMPIat and HMPIlsr with iPromoter-2L [26] on the Escherichia coli K-12 datasets. Table 7 shows the outcomes of identifying promoter subtypes through the HMPI, HMPIat, HMPIlsr and iPromoter-2L methods on the Escherichia coli K-12 dataset, which are indicated in Table 2.

**Table 7:** Comparison of the performance of the HMPI, HMPIat, HMPIlsr, and iPromoter-2L on identifying subtypes of *Escherichia coli* K-12

Organism	Subtype	iPromoter-2L		HMPI		HMPIat		HMPIlsr	
		*Acc* (%)	*Mcc*	*Acc* (%)	*Mcc*	*Acc* (%)	*Mcc*	*Acc* (%)	*Mcc*
*Escherichia coli* K-12	σ^24^	93.50	0.7338	95.45	0.8443	94.76	0.8138	**96.85**	**0.8901**
	σ^28^	96.82	0.5708	97.20	0.6547	**97.55**	**0.7078**	97.20	0.6777
	σ^32^	94.41	0.6524	93.71	0.6343	94.76	0.6855	**95.10**	**0.7100**
	σ^38^	**94.69**	0.2962	94.41	0.2644	94.41	**0.3782**	94.06	0.2219
	σ^54^	94.04	**0.6459**	96.50	0.2616	**96.85**	0.3196	96.15	0.2531
	σ^70^	80.66	0.6056	85.66	0.7037	86.01	0.7117	**86.36**	**0.7188**

The best results are shown in bold

According to the comparison in Table 7, HMPIlsr achieves the best results and outperforms iPromoter-2L [26] on both the $Acc$ and $Mcc$ indices on three subtypes of σ^24^, σ^32^ and σ^70^, which demonstrates that the HMPIlsr model with improved labelling smoothing regularization achieves good performance at identifying prokaryotic promoter subtypes. Besides, HMPIat achieves the best results on both the $Acc$ and $Mcc$ indices on the σ^28^ subtype, outperforming iPromoter-2L by 13.7% on the $Mcc$; achieves the best $Mcc$ on the σ^38^ subtype; and achieves the best $Acc$ on σ^54^. This demonstrates that the HMPIat model with the improvement of the ADASYN method and transfer learning has advantages in enhancing the identification performance of subtypes with small data volumes. In summary, the experiments in this section indicate that the HMPI performs very well at identifying prokaryotic promoters, and the improved HMPI models achieve good results in identifying subtypes of prokaryotic promoters. It is further demonstrated that the hybrid HMPI model is effective at identifying promoters.

## Discussion

Table 5 demonstrates the validity of the HMPI at identifying eukaryotic promoters when compared to several existing methods. Besides, according to Tables 6 and 7, the HMPI performs very well at identifying prokaryotic promoters, and the improved HMPI models achieve good results in identifying subtypes of prokaryotic promoters. We attribute these results to the framework and detail settings of the HMPI. The PSFN in the HMPI utilizes three CNNs blocks to capture fine-grained small-scale local characteristics, the medium-grained features and the larger local features of promoter sequences respectively, and incorporate the centre loss as a portion of the categorization loss function to achieve both intraclass compactness and interclass dispersion. The DSPN in the HMPI is equipped with direct connections which make it has the potential to increase promoter structural profile utilization and enhance information flow.

In addition, HMPI also outperforms both DSPN and PSFN, comparing Table 5 to Tables 3 and 4. The findings suggest that the deduced information implied in structural features may complement the information implied in sequence features in the identification problem of promoters.

## Conclusion

It is critical to correctly identify promoters in order to continue understanding genomic regulatory networks. In the current paper, we developed the HMPI, a hybrid deep learning model for the identification of promoters, which is able to model the structural profiles of promoters and original sequences of promoters simultaneously to comprehensively identify promoters. To derive the features from the original sequences, we first introduce the PSFN, an approach that utilizes and enhances CNNs by incorporating the centre loss as a portion of the categorization loss function to achieve both intraclass compactness and interclass dispersion. Furthermore, we developed the DSPN, a fully connected network with direct links among multiple layers, to represent the structural features of promoters. Since the network is equipped with direct connections, it may be significantly deeper, more efficient and valid; and this network has the potential to increase promoter structural profile utilization and enhance information flow. Finally, we developed the HMPI, a hybrid architecture that combines the DSPN and PSFN to precisely identify promoters. The HMPI can be extended to additional models and features, and it could also be utilized for various biological functional sequences. The HMPI was applied to human, plant and Escherichia coli K-12 strain datasets, and the results showed that the HMPI was successful at extracting the features of promoters while greatly enhancing the performance of identifying promoters on both eukaryotic and prokaryotic datasets. In addition, after improving synthetic sampling, transfer learning and label smoothing regularization, the improved HMPI models achieved good results at identifying subtypes of promoters on prokaryotic promoter datasets.

## Methods

### The framework of the HMPI

Recently, studies employing original sequences have yielded promising results in terms of the identification of promoters, indicating that original promoter sequences might include additional discriminative details compared to signal features recovered from recognized functional regions [14, 15]. We propose the PSFN method to extract promoter sequence characteristics and model the original promoter sequences based on this assumption. Quadrature encoding is employed for encoding promoter sequences, and the class label is turned into a class centre vector using an embedding layer to calculate the centre loss.

Furthermore, when compared to coding or nonregulatory sequences, investigations have demonstrated that promoters do have distinct structural profiles, and the sequence itself is primarily responsible for determining them [18]. The structural profile property matrix was used to generate the structural profiles of dinucleotides, twelve in number, within promoter sequences in this work. We develop the DSPN to extract additional structural traits and details out of these twelve attributes. Because of the relatively straightforward connections among layers, the input structural features and additional front-layer data could be exploited in a better way.

Because promoter sequence features have a better probability of characterizing the information pertaining to promoter elements whereas structural features indicate the structural information of the promoter, we combined the DSPN and PSFN to design the HMPI.

**Fig. 3:**
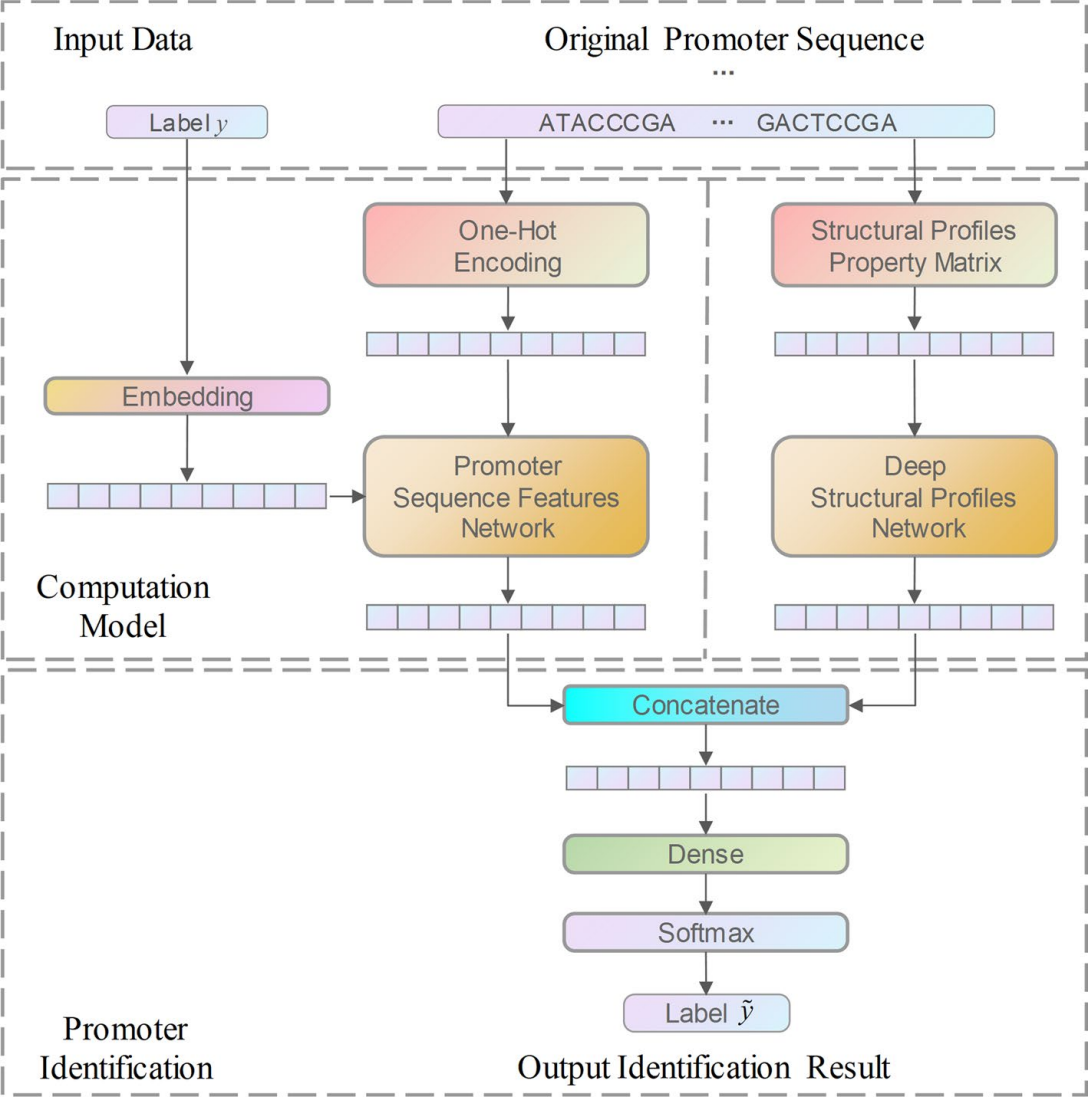
The framework of the HMPI (hybrid model for promoter identification)

Each of the individual sequence traits extracted through the PSFN and the structural traits extracted through the DSPN are concatenated as one novel characteristic in order to illustrate the nonpromoter or original promoter sequences. A completely connected layer (indicated as Dense in Fig. 3) and a softmax layer identify the new features. Figure 3 depicts the overall framework.

### Modelling the original promoter sequences

**Fig. 4:**
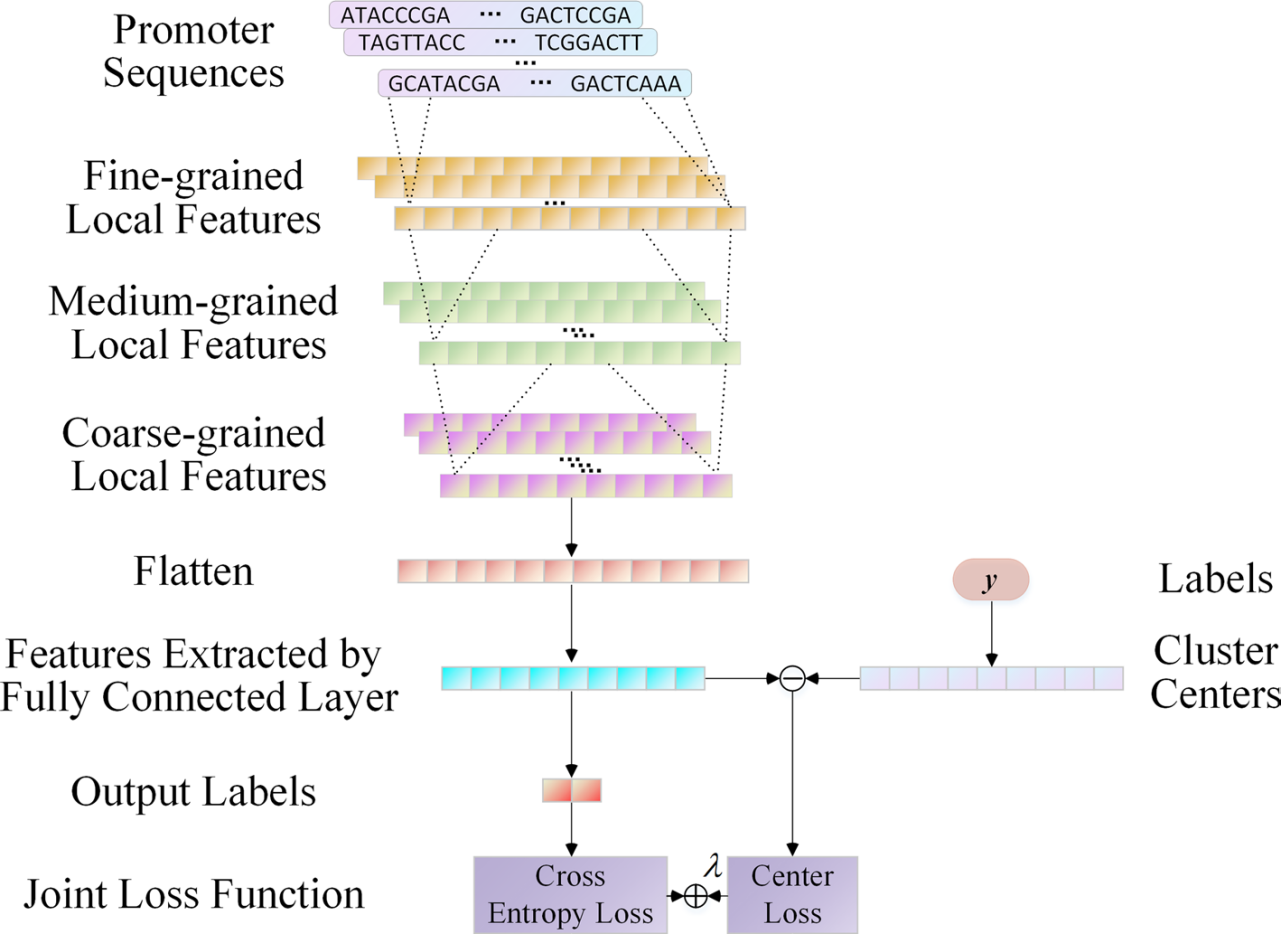
The framework of the PSFN (promoter sequence features network)

The original sequences of the promoter are employed as input for the analysis and extraction of the possible features within the sequence comprising the promoter and to improve the performance in terms of the identification of promoters. We proposed the PSFN method for modelling the original promoter sequences using CNNs and used experiments to confirm its validity. Our inspiration was primarily based on the application of CNNs in promoter categorization and functional gene element analysis [14, 41]. Figure 4 depicts the PSFN methodical framework. The method's details are described as follows.

#### Promoter sequence encoding

To encode promoter sequences, quadrature encoding is employed in an attempt to lose the least possible sequence information during encoding. $Seq = {\text{B}}_{1} {\text{B}}_{{2}} {\text{B}}_{{3}} \cdots {\text{B}}_{j} \cdots {\text{B}}_{L}$, where ${\text{B}}_{j} \in \left( {\text{A,C,G,T}} \right)$ is used to express a sequence of raw genomes of length $L$. Following the one-hot encoding of individual bases, the sequence $Seq$ can be depicted as a 4 × *L* matrix $S$:

3 $$S_{i,j} = \left\{ {\begin{array}{*{20}l} {1,} \hfill & {\quad if\,{\text{B}}_{j} \,is\,the\,ith\,item\,of\,{\text{(A,C,G,T)}}} \hfill \\ {0,} \hfill & {\quad Otherwise} \hfill \\ \end{array} } \right..$$

#### Feature extraction

We make various tweaks to the basic CNNs [42] to minimize the computational complexity and obtain more useful features. A set of three CNNs blocks are used in the PSFN to learn the encoding matrices. A dropout layer, a LeakyReLU activation layer, and a convolution layer compose each block of the CNNs. The CNNs block can be summarized as follows:

4 $$f({\mathbf{x}}) = {\text{Dropout}}({\text{LeakyReLU}}({\text{conv}}({\mathbf{x}})))$$

where ${\mathbf{x}}$ denotes the input of a CNNs block, and ${\text{conv}}({\mathbf{x}})$ denotes the convolution layer. Because the first block is the basis for the follow-up blocks, we utilized one-dimensional convolutional kernels, and the feature mappings act on 4 channels. Thirty-two feature maps comprise the first convolution block for capturing fine-grained and small-scale local characteristics having a slim receptive field, with a kernel size of 3. Sixty-four feature maps comprising a kernel size of 4 constitute the second convolution block for learning the medium-grained features using the first block's feature maps. Within the last convolution block, the size of the kernel is 5 to extract the local features with larger receptive fields. High-level and coarse-grained local features are stored by 128 feature maps. Finally, the last convolution block's output is flattened, and a completely connected layer is utilized to deduce the ultimate sequence characteristics. Later, as a numeric vector, the expression of an original sequence of promoters is possible.

In an attempt to attain a network that is sparser, the ${\text{LeakyReLU}}$ activation function, which aids in accelerating the calculations and alleviating the vanishing gradient problem, is employed as the activation layer [43]. The ${\text{LeakyReLU}}$ function is presented in Additional file 1: Equation S1. To confirm the activity of adequate neurons, the $\alpha$ in the ${\text{LeakyReLU}}$ function is 0.2.

Furthermore, the ${\text{ReLU}}$ is utilized as activation function in the last connected layer. The function is given Additional file 1: Equation S2.

During training, a dropout layer is employed to randomly remove units from the network with a given probability with the goal of preventing units from overadapting [44]. The likelihood of dropping out is set to 0.25 in this case.

#### The loss function

A loss function is employed as the supervisory signal to train a network in the majority of the existing CNNs. Following the feature extraction layer, there is a fully connected layer with $K$ neurons ($K{ = }2$ in a binary classification task, such as promoter identification) that uses the softmax activation function to calculate the probability that the sample is placed in every class:

5 $$p(k) = {\text{Softmax}}({\mathbf{W}}_{k}^{T} {\mathbf{x}} + {\mathbf{b}}_{k} ) = \frac{{e^{{{\mathbf{W}}_{k}^{T} {\mathbf{x}} + {\mathbf{b}}_{k} }} }}{{\sum\nolimits_{i = 1}^{K} {e^{{{\mathbf{W}}_{i}^{T} {\mathbf{x}} + {\mathbf{b}}_{i} }} } }}$$

Herein, the probability that the input belongs to category $k$ is represented via $p(k) \in [0,1], k = 1, \ldots ,K$.

The CCE loss is the most frequently employed softmax loss function and is represented by the following expression:

6 $$L_{CCE} = - \sum\limits_{k = 1}^{K} {\log (p(k))q(k)}$$

In this equation, the distribution of the ground truth is denoted by $q(k)$, the output class is represented by *k* and the actual class is $y$.

7 $$q(k) = \left\{ {\begin{array}{*{20}l} {0,} \hfill & {\quad k \ne y} \hfill \\ {1,} \hfill & {\quad k = y} \hfill \\ \end{array} } \right.$$

Then Eq. (6) is equivalent to Eq. (8).

8 $$L_{CCE} = - \log (p(y))$$

The softmax loss function $L_{CCE}$ decreases whereas and the interclass dispersion increases as model training progresses. The centre loss is utilized as a portion of the loss function within CNNs to improve the discriminative capability of the modelling effect [45]. We may prepare CNNs to attain features possessing two primary learning objectives, intraclass compactness and interclass dispersion, simultaneously using the combined supervision of the centre loss and softmax loss.

The cluster centre is ascertained using the real class of a specific sample feature, and the centre loss is given by the Euclidean distance among the cluster centres and the sample features. The joint loss function is shown in the diagram below.

9 $$L_{CCE - CL} = - \log (p(y)) + \lambda \left\| {{\varvec{x}} - {\varvec{c}}_{y} } \right\|^{2}$$

Herein, the sample feature is denoted by ${\varvec{x}}$, and the cluster centre of class $y$ is denoted by ${\varvec{c}}_{y}$. The label of the class is converted into the vector of class centre ${\varvec{c}}_{y}$ with the identical length as ${\varvec{x}}$ using an embedding layer.

### Extraction of the structural characteristics of promoters

Structural profile properties refer to specific characteristics of DNA molecules, such as their stability and bendability, which are related to dynamic DNA structure (potential to change in conformation) [16]. Although the nucleotide sequence mostly determines these structural profile properties, research has demonstrated that promoters do possess different patterns in these properties compared to other sequences, and these properties play an important role in promoter identification [17, 18]. The values of twelve structural profile properties associated with each dinucleotide are provided in Additional file 1: Table S1.

To obtain the structural characteristics of promoters, we first obtained the structural profile property matrix and calculated the twelve properties of dinucleotides in the structural profiles of promoter sequences. We also conduct normalization (subtract the mean and divide by the standard deviation) for properties to ensure that each property can possess the same opportunity to be calculated. Second, the DSPN was developed to model the twelve considered structural profile qualities and extract promoter structural traits. The following are the specifics.

#### The matrix of structural profile properties

For each of the sixteen combinations of dinucleotides that include ${\text{AA}}$,${\text{AC}}$,${\text{AG}}$,${\text{AT}}$,${\text{CA}}$,$\ldots$, and ${\text{TT}}$ in a DNA sequence, there are various structural profile ($SP$) properties. Herein, the twelve $SP$ properties listed as follows [46] have been implemented: (1) $SP1$: A-philicity [47], (2) $SP2$:base stacking [48], (3)$SP3$:B-DNA twist [49], (4)$SP4$:bendability [50], (5)$SP5$: bending stiffness of DNA [51], (6)$SP6$: denaturation of DNA [52], (7)$SP7$:duplex disrupt energy [53], (8)$SP8$:duplex free energy [54], (9)$SP9$:propeller twist [49], (10)$SP10$: deformation of protein [55], (11)$SP11$: twist of protein-DNA [55], and (12) $SP12$:Z-DNA [56]. Additional file 1: Table S1 has the original values for each of these twelve attributes for each dinucleotide. We standardized the original values of the twelve characteristics because of their various distributions. The following is the normalization equation:

10 $$SPi_{k}^{std} = \frac{{SPi_{k} - mean_{K} \left( {SPi} \right)}}{{std_{K} \left( {SPi} \right)}}$$

As mentioned above, for every dinucleotide, such as ${\text{AA}}$,${\text{AC}}$,${\text{AG}}$,${\text{AT}}$,${\text{CA}}$,$\ldots$, and ${\text{TT}}$(a total of 16 combinations), there are 12 $SP$ properties. Herein, $SPi_{k}^{{}}$ denotes the value of the ith ($i = 1,2, \cdots ,12$)$SP$ property for the $k{\text{ - th}}$($k = 1,2, \cdots ,16$) combination of dinucleotides. In addition, the normalized $SPi_{k}^{{}}$ is represented by $SPi_{k}^{std}$. Furthermore, $mean_{K} \left( {SPi} \right)$ represents the mean and $std_{K} \left( {SPi} \right)$ represents the standard deviation of $SPi$ for the $K{\text{ - th}}$ combination of dinucleotides.

We apply a window 2 bp long on the raw promoter sequence of length *L* to obtain the matrix of structural profile properties, and the dinucleotides within the window were represented with twelve normalized values. Finally, we can attain the $12 \times( L - 1)$ matrix of structural profile properties. As an example, a length *L* promoter sequence $Seq = {\text{B}}_{1} {\text{B}}_{{2}} {\text{B}}_{{3}} \cdots {\text{B}}_{j} \cdots {\text{B}}_{L}$, ${\text{B}} \in \left\{ {{\text{A,C,G,T}}} \right\}$ can be presented as a matrix $S$ like the one presented below.

11 $$S_{i,j} = SPi_{{}}^{std} \left( {{\text{B}}_{j} {\text{B}}_{j + 1} } \right)$$

Herein, $SPi_{{}}^{std} \left( {{\text{B}}_{j} {\text{B}}_{j + 1} } \right)$ defines the $i{\text{ - th}}$ ($i = 1,2, \cdots ,12$) normalized $SP$ value corresponding to dinucleotide ${\text{B}}_{j} {\text{B}}_{j + 1}$.

#### The DSPN (deep structural profiles network)

**Fig. 5:**
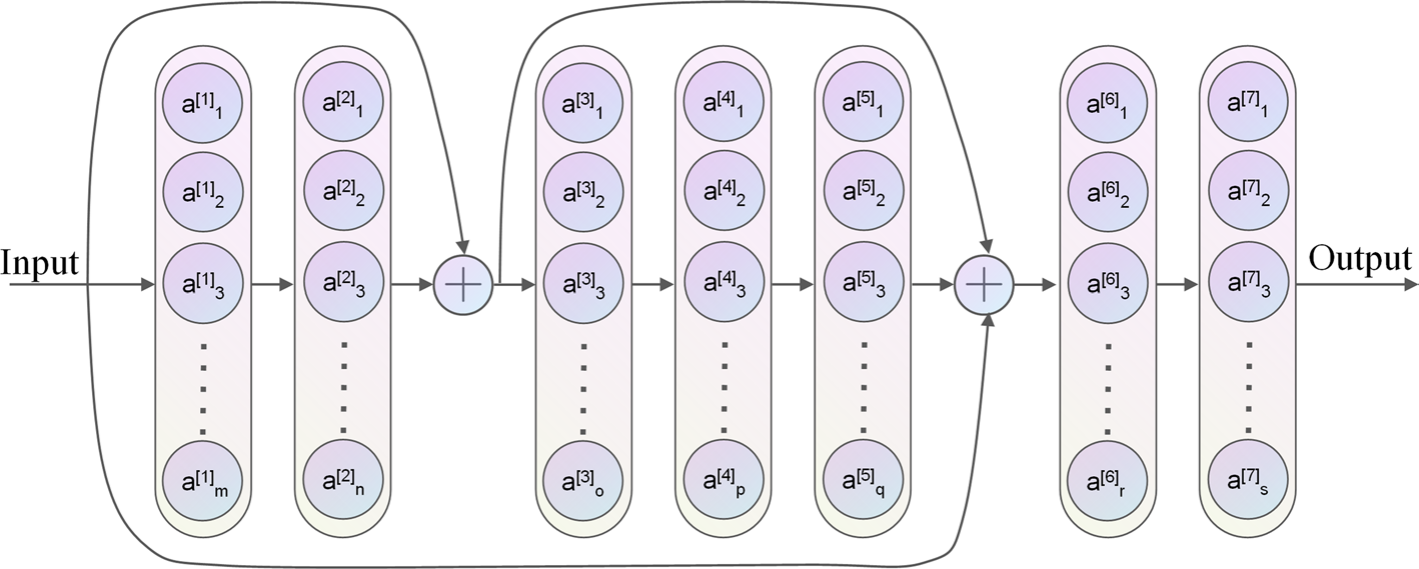
The framework of the DSPN (deep structural profiles network)

The DSPN uses DenseNet [21] and a fully connected network to represent the considered promoter structural profiles. We develop the DSPN, which has partial direct connections among layers, for the further modelling of the calculated structural properties and for extracting structural features. Several layers are furnished with uninterrupted attainment to link the gradients directly from the loss function with the input structural attributes. These direct connections ensure a more desirable flow of information and use of the structural profiles of promoters. Furthermore, the links in the network produce short routes, which help to alleviate the vanishing gradient issue, encourage reuse of features, and make the network relatively easy to train. The DSPN framework is shown in Fig. 5.

In the DSPN, seven blocks are employed, as seen in Fig. 5. A fully connected layer, a LeakyReLU activation layer, a dropout layer, and a normalization layer make up individual blocks. The following is a description of each block.

12 $$f({\mathbf{x}}) = {\text{BatchNorm}}\left( {{\text{Dropout}}({\text{LeakyReLU}}({\text{Dense}}({\mathbf{x}})))} \right)$$

In this equation, ${\text{Dense}}({\mathbf{x}})$ represents the fully connected layer.

Moreover, the ${\text{LeakyReLU}}$ activation function is utilized in the activation layer to obtain a better sparse network. Then, a dropout layer is utilized to drop units at random at a probability of 0.2. In the normalization layer, batch normalization (shown in Additional file 1: Equation S3) is used to reproduce the distribution, strengthening the training process stronger and enhancing the training accuracy.

The structural properties of *SPs* are concatenated as part of the inputs of blocks 3 and 6. In addition, the features extracted by block 2 are linked to block 6 due to the direct linkages. Furthermore, because certain blocks are directly linked to the gradients, the vanishing gradient problem is mitigated to a certain extent. In the seven DSPN blocks, the number of neurons in the connected networks is set to 250, 1000, 250, 1000, 1500, 1000, and 128, respectively. The output of the 7th block is a vector that represents the extracted structural feature of the primary promoter sequence, which is fed in as the matrix of the properties of structural profiles.

In DSPN, a fully connected layer containing two nodes follows the extracted structural features and outputs the probability that the sample sequence belongs to each category utilizing a softmax activation function (see Eq. 5). The CCE loss is the loss function used in the DSPN (see Eq. 8).

## Supplementary Information

**Additional file 1.** Supplementary Material for a Successful Hybrid Deep Learning Model aiming at Promoter Identification.

## Data Availability

The datasets generated and analyzed during the current study are available at https://github.com/YingWang-SEI/HMPI.

## Abbreviations

TSS: Transcription start site
HMPI: Hybrid model for promoter identification
PSFN: Promoter sequence features network
DSPN: Deep structural profiles network
CNNs: Convolutional neural networks
EPD: Eukaryotic promoter database
*Sn*: Sensitivity
*Sp*: Specificity
*Mcc*: Matthew correlation coefficient
*Acc*: Accuracy
PSFNcce: The PSFN utilizing categorical cross-entropy as loss function
PCA: Principal component analysis
TSNE: T-distributed stochastic neighbor embedding
CCE: Categorical cross entropy
$$SP$$: Structural profile
PSEKNC: Pseudonucleotide composition
HMPIat: The HMPI with adaptive synthetic sampling approach
HMPIlsr: The HMPI with a label smoothing regularization
