# Supplementary material for: A successful hybrid deep learning model aiming at promoter identification
Source: BMC Bioinformatics. 2022 May 31;23(Suppl 1):206. doi: 10.1186/s12859-022-04735-6 (PMC9158169; doi:10.1186/s12859-022-04735-6)
Supplement: Supplementary file 1 — Additional file 1. Supplementary Material for a Successful Hybrid Deep Learning Model aiming at Promoter Identification. [file 12859_2022_4735_MOESM1_ESM.docx]

Supplementary Material for a Successful Hybrid Deep Learning Model aiming at Promoter Identification

# Supplementary to Experiments and Results:


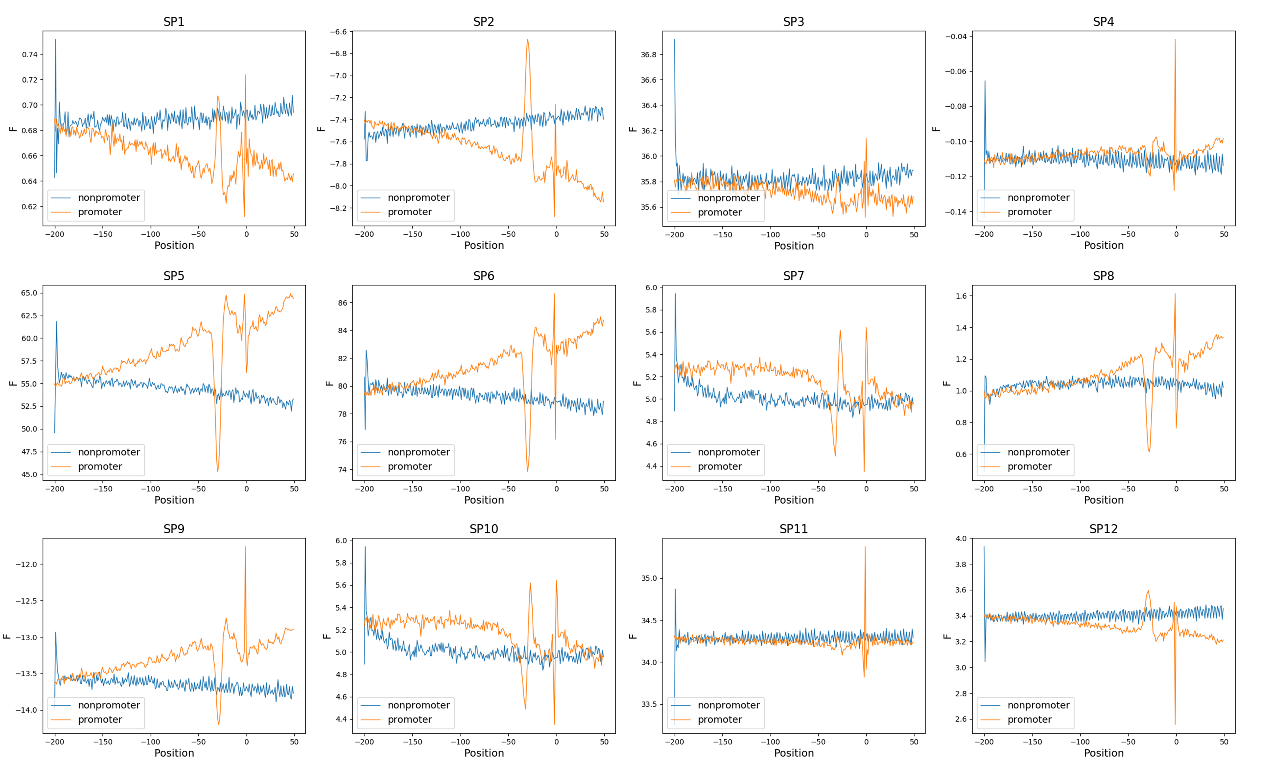


Figure S1 Statistical Analysis Results of the Twelve$SP$Properties on Plant Datasets.

In Figure S1, there are twelve statistical analysis figures for the twelve $SP$ properties respectively. In each figure, the side coordinate represents the base position of dinucleotide, where the TSSs locate at side coordinate 0. And the longitudinal coordinate represents the mean of the corresponding property $SPi$ for dinucleotides from plant promoters or nonpromoters.

# Supplementary to Methods:

The activation function$\text{LeakyReLU}$is presented in Equation S1.

$\text{LeakyReLU}(x)=\left\{\begin{matrix} x,\text{ }if\text{ }x\geq0 \\ \alpha x,\text{ }if\text{ }x\leq0 \end{matrix}\right.$ Equation S1

The activation function$\mathrm{ReLU}$is presented in Equation S2.

$\text{ReLU}(x)=\left\{\begin{matrix} x,\text{ }if\text{ }x\geq0 \\ 0,\text{ }if\text{ }x\leq0 \end{matrix}\right.$ Equation S2

The description of Batch Normalization is presented in Equation S3.

$\left\{\begin{matrix} \mu\text{=}\frac{1}{m}\sum^{m} x_{i} \\ \sigma^{2}=\frac{1}{m}\sum^{m} (x_{i}-\mu)^{2} \\ x_{i}^{norm}=\frac{x_{i}-\mu}{\sqrt{\sigma^{2}+\varepsilon}}(\mathrm{mean}=0,\mathrm{var}=1) \\ \tilde{x}_{i}=\gamma x_{i}^{norm}+\beta\end{matrix}\right.$ Equation S3

herein the $i\text{-th}$ neuron’s input is denoted by $x_{i}$, the size of the batch is given by $m$, the variance and mean of the batch are respectively represented by $\sigma^{2}$ and $\mu$. $x_{i}^{norm}$represents the value of $i\text{-th}$ neuron of the standard Gaussian distribution. The $i\text{-th}$ output of the normalized batch distribution of reconstruction is given by $\tilde{x}_{i}$. $\varepsilon$ is a value of negligible magnitude added to prevent the denominator from becoming zero. Besides, $\gamma$ and $\beta$ are a pair of parameters pertaining to reconstruct distribution.

Table S1 The Original Values of The Twelve *SP* Properties for Each Dinucleotide

|  |  | $SPi$($i=1,2,\cdots,12$) | | | | | | | | | | | |
| --- | --- | --- | --- | --- | --- | --- | --- | --- | --- | --- | --- | --- | --- |
|  | **Code** |  |  |  |  |  |  |  |  |  |  |  |  |
| $k\text{=}1$ | **AA** | 0.97 | -5.37 | 35.5 | -0.27 | 35 | 66.51 | 12.1 | -1.2 | -18.66 | 12.1 | 35.1 | 3.9 |
| $k\text{=2}$ | **AC** | 0.13 | -10.5 | 33.1 | -0.21 | 60 | 108.8 | 9.8 | -1.5 | -13.1 | 9.8 | 31.5 | 4.6 |
| $k\text{=3}$ | **AG** | 0.33 | -6.87 | 30.6 | -0.08 | 60 | 85.12 | 6.3 | -1.5 | -14 | 6.3 | 31.9 | 3.4 |
| $k\text{=4}$ | **AT** | 0.58 | -6.57 | 43.2 | -0.28 | 20 | 72.29 | 2.1 | -0.9 | -15.01 | 2.1 | 29.3 | 5.9 |
| $k\text{=5}$ | **CA** | 1.04 | -6.57 | 37.7 | -0.01 | 60 | 64.92 | 6.1 | 1.9 | -9.45 | 6.1 | 37.3 | 1.3 |
| $k\text{=6}$ | **CC** | 0.19 | -8.26 | 35.3 | -0.03 | 130 | 99.31 | 2.9 | 3.1 | -8.11 | 2.9 | 32.9 | 2.4 |
| $k\text{=7}$ | **CG** | 0.52 | -9.69 | 31.3 | -0.03 | 85 | 88.84 | 4.5 | 3.6 | -10.03 | 4.5 | 36.1 | 0.7 |
| $k\text{=8}$ | **CT** | 0.33 | -6.78 | 30.6 | -0.18 | 60 | 85.12 | 1.6 | 1.6 | -14 | 1.6 | 31.9 | 3.4 |
| $k\text{=9}$ | **GA** | 0.98 | -9.81 | 39.6 | 0.03 | 60 | 80.03 | 2.3 | 1.6 | -13.48 | 2.3 | 36.3 | 3.4 |
| $k\text{=10}$ | **GC** | 0.73 | -14.6 | 38.4 | 0.02 | 85 | 135.8 | 4 | 3.1 | -11.08 | 4 | 33.6 | 4 |
| $k\text{=11}$ | **GG** | 0.19 | -8.26 | 35.3 | -0.06 | 130 | 99.31 | 6.1 | 3.1 | -8.11 | 6.1 | 32.9 | 2.4 |
| $k\text{=12}$ | **GT** | 0.13 | -10.51 | 33.1 | -0.18 | 60 | 108.8 | 2.1 | 1.3 | -13.1 | 2.1 | 31.5 | 4.6 |
| $k\text{=13}$ | **TA** | 0.73 | -3.82 | 31.6 | 0.18 | 20 | 50.11 | 2.3 | 1.5 | -11.85 | 2.3 | 37.8 | 2.5 |
| $k\text{=14}$ | **TC** | 0.98 | -9.81 | 39.6 | -0.11 | 60 | 80.03 | 4.5 | 1.6 | -13.48 | 4.5 | 36.3 | 3.4 |
| $k\text{=15}$ | **TG** | 1.04 | -6.57 | 37.7 | 0.13 | 60 | 64.92 | 9.8 | 1.9 | -9.45 | 9.8 | 37.3 | 1.3 |
| $k\text{=16}$ | **TT** | 0.97 | -5.37 | 35.5 | -0.28 | 35 | 66.51 | 2.8 | 1.9 | -18.66 | 2.8 | 35.1 | 3.9 |
